# Granulomatosis with Polyangiitis Manifesting as a Symptomatic Sellar Mass in a Young Woman

**DOI:** 10.7759/cureus.5823

**Published:** 2019-10-02

**Authors:** Sachin P Gadani, Rohini Nadgir, Annika Windon, Lisa Rooper, Fahid Alghanim

**Affiliations:** 1 Neurology, Johns Hopkins Hospital, Baltimore, USA; 2 Radiology, Johns Hopkins Hospital, Baltimore, USA; 3 Pathology, Johns Hopkins Hospital, Baltimore, USA; 4 Pulmonary and Critical Care, University of Maryland, Baltimore, USA

**Keywords:** gpa, granulomatosis with polyangiitis, wegner's granulomatosis, pituitary mass, hypopituitarism, neuroimmunology, pituitary pathology

## Abstract

A pituitary mass is a rare and poorly understood complication of granulomatosis with polyangiitis (GPA). Here we describe the case of a young woman with GPA who presented with signs and symptoms initially suggestive of meningitis but was ultimately found to have hypopituitarism and an enlarging sellar mass. She underwent transsphenoidal biopsy, which revealed an abundance of sterile inflammation and necrosis consistent with GPA-related inflammation. This case demonstrates a rare complication of GPA, i.e., a pituitary mass, initially mimicking meningitis. GPA-related pituitary involvement has an unknown pathogenesis and can have debilitating long-term consequences including chronic hypopituitarism and vision impairment, highlighting the need for further research.

## Introduction

Granulomatosis with polyangiitis (GPA) is an ANCA (anti-neutrophilic cytoplasmic autoantibody) associated autoimmune vasculitis commonly involving the upper respiratory tract, lungs, and kidneys, but having the potential to affect nearly any tissue including the nervous system. In one study of 128 patients with GPA, 64 (50%) had nervous system involvement, though the majority (56 patients) of them had only peripheral nerve disease [1]. In the central nervous system (CNS), GPA can rarely cause cranial nerve palsies, pachymeningitis, CNS vasculitis, or pituitary lesions [2]. Pituitary lesions are exceedingly uncommon; in a study of 819 patients with GPA, only 9 (1.1%) had pituitary involvement [3]. We identified 57 reported cases of GPA with pituitary involvement in the literature to date, with diabetes insipidus presenting often and panhypopituitarism presenting as a less common complication [3-8]. Here we describe the case of a young woman with GPA who presented with symptoms initially suggestive of meningitis, but on further evaluation had hypopituitarism and was found to have an enlarging pituitary mass caused by GPA. We will describe the course of her diagnosis, including imaging and pathology, initial management, and response. We will conclude by discussing this case in the context of other published reports and by speculating on the reason why GPA might target the pituitary while infrequently affecting the CNS parenchyma elsewhere.

## Case presentation

A 25-year-old woman with GPA, chronic ear and sinus infection, and a known sellar mass presented to the emergency department with recurrent headache and vomiting (Poster: Sachin Gadani, Fahid Alghanim, Annika Windon, Lisa Rooper, Rohini Nadgir, and Karan Desai. Granulomatosis with polyangiitis manifesting as a symptomatic sellar mass in a young woman. Society of General Internal Medicine; 5/2019). GPA had been diagnosed one year prior through sinus biopsy, and she has been maintained on prednisone and methotrexate. At the time, she was known to have upper respiratory tract involvement mainly within her sinuses and ears. The day prior to admission, she developed a left-sided headache and subjective fever followed by nausea and profuse vomiting. She stated that these symptoms, with the exception of fever, were consistent with her typical GPA flares, which she had been experiencing weekly for the past several months. Rarely the symptoms did not resolve and she would present to the emergency department, with her last presentation assumed to be a GPA flare and managed successfully with diphenhydramine and methylprednisolone.

During examination, the patient also revealed eight months of amenorrhea, fatigue, cold intolerance, 20 lb of weight gain, and constipation. She was febrile to 38.3°C with otherwise normal vital signs. Physical examination showed no meningismus, papilledema, or visual field deficit. She refused lumbar puncture because the symptoms were similar to those of her regular GPA flares. Blood cultures were negative. Thyroid function studies showed a TSH level of 0.06 uIU/mL (0.4-4.0), free T4 level of 0.4 ng/dL (0.9-2.4), and total T3 level of 0.49 ng/mL (2.3-6.2), indicating central hypothyroidism. Further testing showed a low prolactin level of 0.1 ng/mL (2-29), luteinizing hormone level of 0.4 mIU/mL (0.5-76.3), and follicle-stimulating hormone (FSH) level of 2.5 mIU/mL (15-20), consistent with central hypogonadism. Insulin-like growth factor-1 (IGF-1) was within normal range at 108 ng/mL (63-373). Morning cortisol was measured as 0.7 ng/dL (10-20) but confounded by chronic prednisone use. There was no clinical evidence of anti-diuretic hormone (ADH) deficiency.

**Figure 1:**
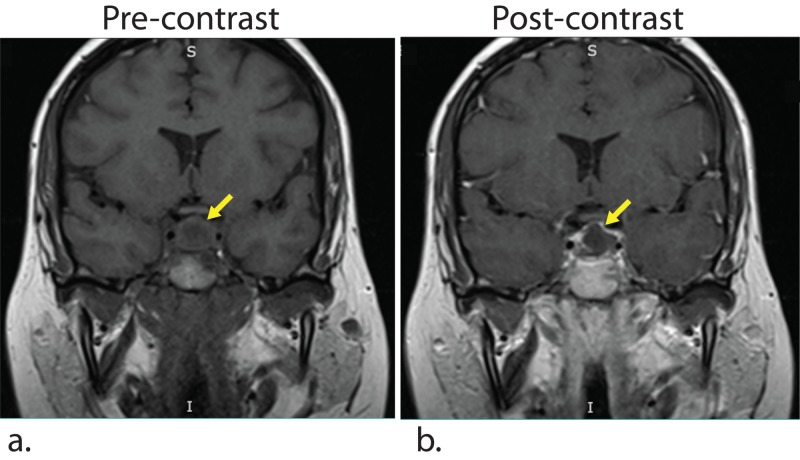
MRI 10 months prior to admission (a) Pre-contrast T1-weighted MRI image demonstrates a 1.2 x 1.6 x 1.5 cm (AP x TR x CC) lesion in the sella turcica, which is thought to be Rathke’s cleft cyst (yellow arrow). (b) Post-contrast T1-weighted MRI image shows lack contrast enhancement. AP, anteroposterior; TR, transverse; CC, craniocaudal.

**Figure 2:**
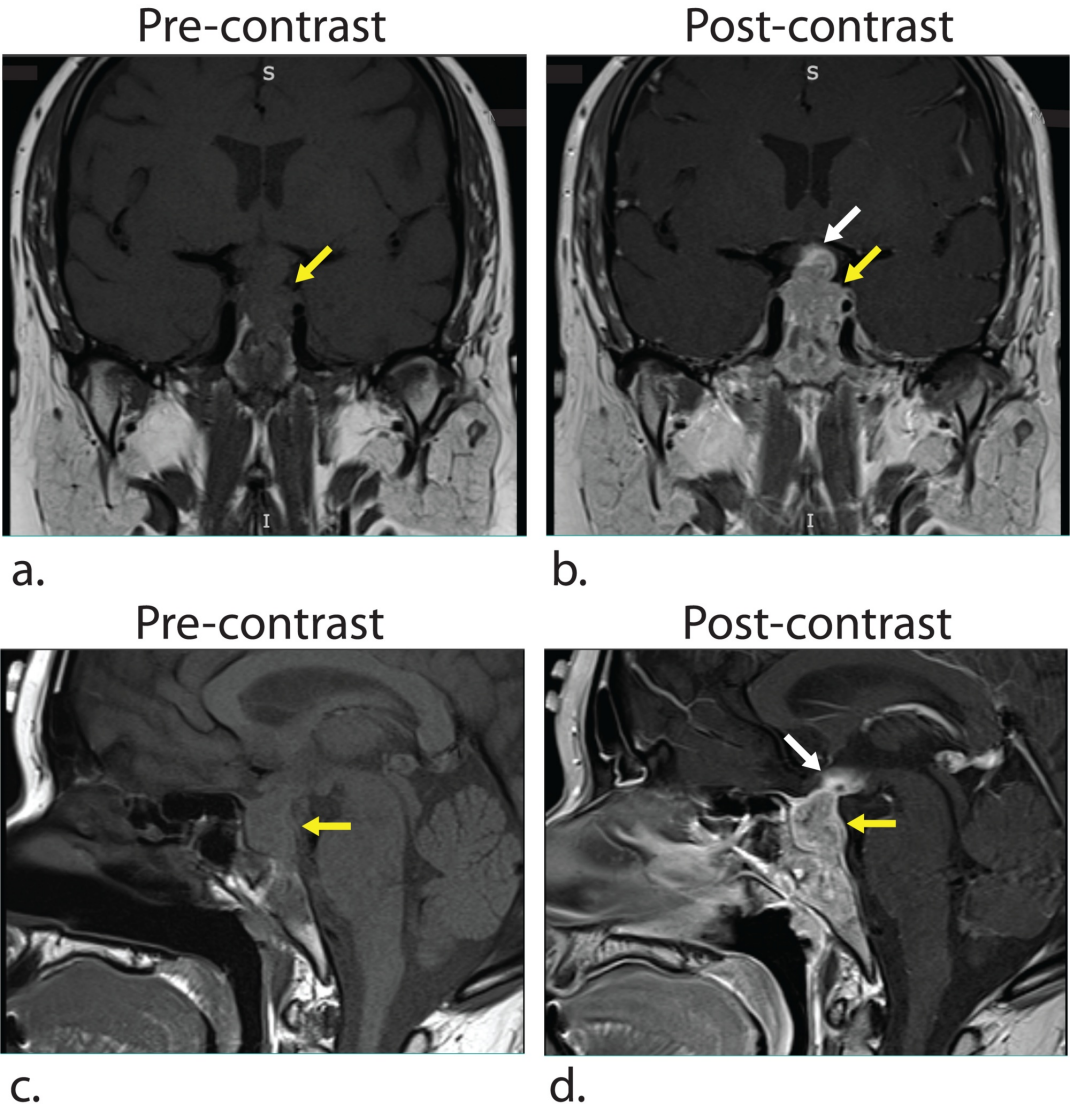
Repeat MRI showed interval enlargement and contrast enhancement of the lesion Pre-contrast T1-weighted coronal (a) and sagittal (c) MRI images demonstrate a 1.5 x 1.6 x 2.4 cm (AP x TR x CC) lesion in the sella turcica (yellow arrows) with supra-sellar extension (white arrows). Post-contrast T1-weighted coronal (b) and sagittal (d) MRI images show contrast enhancement of the lesion. AP, anteroposterior; TR, transverse; CC, craniocaudal.

Her presentation of recurrent headache, nausea/vomiting, and hypopituitarism led to a further investigation of her known sellar mass. Ten months prior to admission, a cystic, non-enhancing sellar lesion with suprasellar extension was seen incidentally on an MRI performed to investigate mastoiditis (Figure 1). At that time, it measured 1.2 x 1.6 x 1.5 cm (AP x TR x CC [anteroposterior x transverse x craniocaudal]), did not cause mass effect, and was thought to likely represent Rathke’s cleft cyst. A repeat MRI scan showed an interval enlargement of the lesion, now 1.5 x 1.6 x 2.4 cm and extending posteriorly into the clivus and superiorly into the hypothalamus (Figure 2). The lesion showed T1 hypointensity, T2 heterogeneity, and mild diffusion restriction, and was notably enhancing internally post-contrast (Figures 2B, 2D). These interval changes were not consistent with Rathke’s cleft cyst, and, instead, possible etiologies considered were insidious infection, pituitary macroadenoma, and GPA-related inflammation.

**Figure 3:**
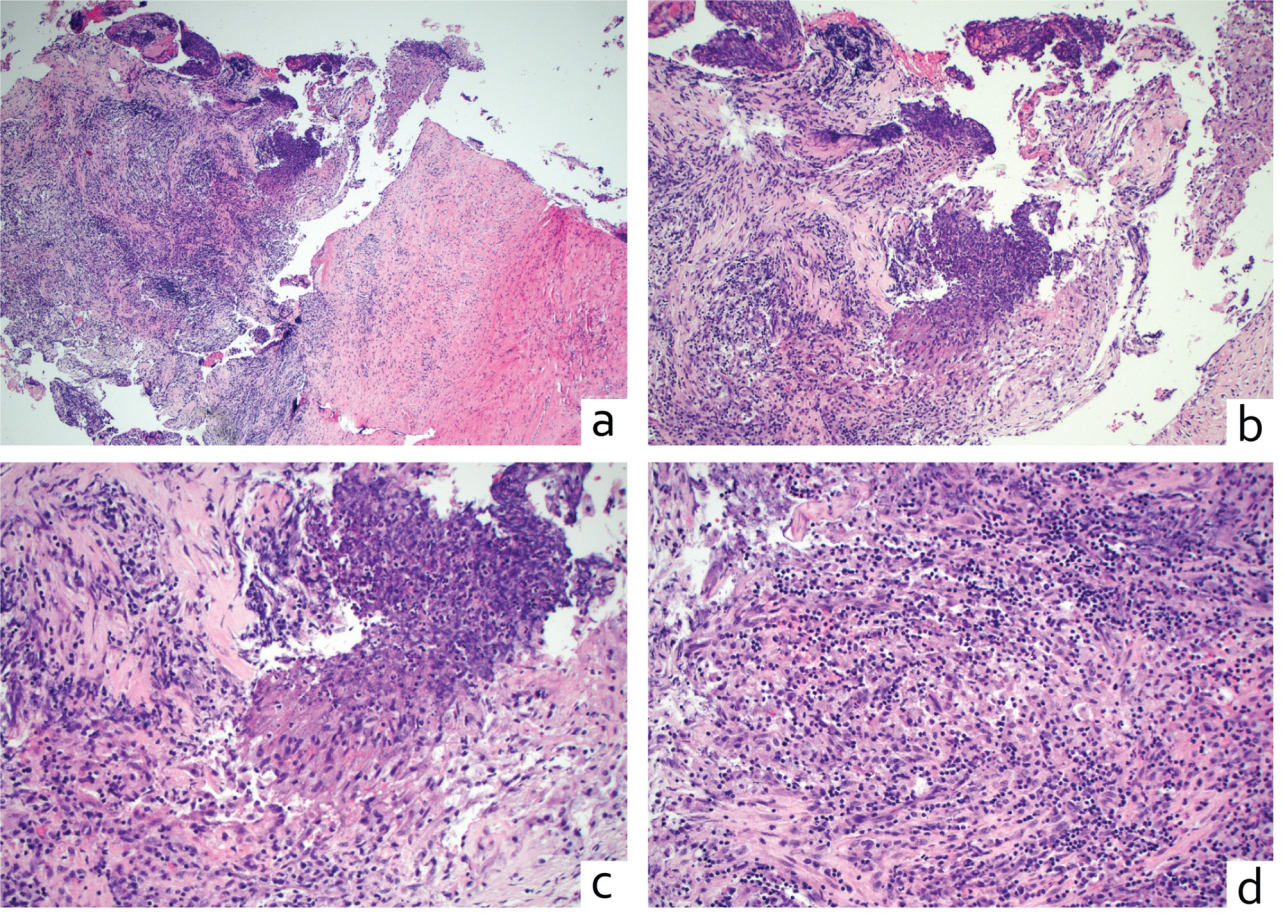
Histopathology of the sellar lesion is consistent with GPA (a) 4x and (b) 10x: H&E sections at a low-power magnification of a sellar mass show fibrous tissue with acute and chronic inflammation and necrosis. (c, d) 20x: H&E sections at an intermediate-power magnification show an admixed macrophage proliferation adjacent to necrosis and chronic inflammation. There are no multinucleated giant cells or epithelioid granulomas observed. GPA, granulomatosis with polyangiitis; H&E, hematoxylin and eosin; AP, anteroposterior; TR, transverse; CC, craniocaudal.

To define the underlying pathology, a transsphenoidal biopsy of the sellar mass and the adjacent bone was performed. Histopathology of the mass demonstrated acute and chronic inflammation. There were prominent reactive macrophages and few PMNs (polymorphonuclear leukocytes) with large areas of fibrosis and necrosis (Figure 3). Gram, PAS, and Giemsa stains were negative for bacterial or fungal organisms, AFB (acid-fast-bacillus) was negative for mycobacteria, and Whipple stain was negative. There was no evidence of vasculitis. Bone histopathology showed evidence of chronic inflammation. Given the prominently activated immune cells without evidence of infection, the most likely diagnosis was autoimmune inflammation related to GPA.

In the immediate postoperative period, she developed central diabetes insipidus, with increased urinary frequency, urgency, and thirst, which did not resolve in the months following surgery. Pituitary hormone deficiency was treated with prednisone, levothyroxine, vasopressin, and a combined oral contraceptive pill. Her presenting symptoms of headache, nausea, and vomiting had significantly improved. She was aggressively immunosuppressed with prednisone 60 mg daily and cyclophosphamide 1 mg/kg (75 mg) daily, with a plan to increase the dose to 2 mg/kg (150 mg). At a dose of 2 mg/kg of cyclophosphamide, she developed mild transaminitis (AST [aspartate transaminase] and ALT [alanine transaminase] elevation), and thus the dose was decreased to 1.25 mg/kg daily. Two months later, the patient requested to be transitioned from cyclophosphamide to rituximab due to concerns regarding fertility. She was weaned from cyclophosphamide and has been on a slow steroid taper and rituximab. At her last clinic visit, she was without any significant GPA flare or progression of pituitary involvement for nine months after initiating rituximab. She continued to have occasional headaches, sinus pressure, and fatigue.

## Discussion

Here we report the case of a young woman with GPA who presented with recurrent headache in the setting of signs, symptoms, and biochemical markers consistent with hypopituitarism and was ultimately found to have a symptomatic GPA-related sellar mass. A challenging aspect of the case was the initial diagnosis of Rathke’s cleft cyst based on the first MRI, which might have delayed the accurate diagnosis of her pituitary lesion. Previously reported cases of pituitary GPA have had highly varied MRI findings, with T1 hypo- or hyperintensity, T2 hyperintensity, and heterogeneous enhancement seen [3, 9-10]. This degree of variability is typical for granulomatous diseases, and imaging findings can often be confused with malignancy [11]. This case sheds light on temporal imaging changes that such a lesion can undergo as it progresses, and underscores the non-specific nature of imaging findings.

Previous reports suggest that pituitary GPA lesions are often amenable to immunosuppression. In one case series of eight GPA patients with pituitary involvement, all achieved remission after induction therapy with corticosteroids combined with either cyclophosphamide or rituximab [9]. In another series of nine patients, all but one achieved remission after corticosteroids combined with secondary immunosuppression [3]. Though several regimens have been used with reported success, the effective regimen has to be found empirically for each patient. One recent case reported rapid disease progression after rituximab was started due to fertility concerns, requiring returning to cyclophosphamide treatment [7], and another case reported a failure of cyclophosphamide with a later response to rituximab [9]. The current case adds another example of the efficacy of cyclophosphamide and rituximab, which both appeared to have successfully suppressed her pituitary inflammation. 

GPA rarely affects the CNS, and when it does, it mostly causes cranial nerve palsy, meningitis, or vasculitis. In the CNS parenchyma, it mostly affects the pituitary, though the reason for this preference remains uncertain. In the biopsy specimen of our case, no vasculitis was seen, possibly suggesting an alternate origin of immune cells. Interestingly, autoimmune pituitary masses have been described independent of GPA, termed as lymphocytic or granulomatous primary hypophysitis. Perhaps, when patients with GPA have pituitary involvement, it may represent idiopathic hypophysitis coexisting with GPA in a patient with a predilection towards autoimmunity. The mechanism of pituitary autoinflammation is altogether uncertain, and it remains to be seen whether it is initiated by autoantibody, autoreactive lymphocytes, or a possible unidentified chronic infection. Detailed histopathologic studies of biopsy specimens may provide further insight into these possibilities.

## Conclusions

In summary, this case report adds to the literature on pituitary GPA, in particular showing the misleading way it may present. Further clinical and basic science research is needed to clarify its pathology, diagnosis, and optimal treatment.
